# A One Health Perspective on *Salmonella* *enterica* Serovar Infantis, an Emerging Human Multidrug-Resistant Pathogen

**DOI:** 10.3201/eid3004.231031

**Published:** 2024-04

**Authors:** Jennifer Mattock, Marie Anne Chattaway, Hassan Hartman, Timothy J. Dallman, Anthony M. Smith, Karen Keddy, Liljana Petrovska, Emma J. Manners, Sanelisiwe T. Duze, Shannon Smouse, Nomsa Tau, Ruth Timme, Dave J. Baker, Alison E. Mather, John Wain, Gemma C. Langridge

**Affiliations:** University of East Anglia, Norwich, UK (J. Mattock, E.J. Manners, A.E. Mather, J. Wain);; UK Health Security Agency, London, UK (M.A. Chattaway, H. Hartman, T.J. Dallman);; National Institute for Communicable Diseases, Johannesburg, South Africa (A.M. Smith, S. Smouse, N. Tau);; University of Pretoria, Pretoria, South Africa (K. Keddy);; Animal and Plant Health Agency, Addlestone, UK (L. Petrovska);; University of the Witwatersrand, Johannesburg (S.T. Duze);; US Food and Drug Administration, College Park, Maryland, USA (R. Timme);; Quadram Institute Bioscience, Norwich (D.J. Baker, A.E. Mather, J. Wain, G.C. Langridge)

**Keywords:** *Salmonella* *enterica* serovar Infantis, bacteria, eBG31, antimicrobial resistance, multidrug resistance, pESI, One Health

## Abstract

*Salmonell*a enterica serovar Infantis presents an ever-increasing threat to public health because of its spread throughout many countries and association with high levels of antimicrobial resistance (AMR). We analyzed whole-genome sequences of 5,284 *Salmonella* Infantis strains from 74 countries, isolated during 1989–2020 from a wide variety of human, animal, and food sources, to compare genetic phylogeny, AMR determinants, and plasmid presence. The global *Salmonella* Infantis population structure diverged into 3 clusters: a North American cluster, a European cluster, and a global cluster. The levels of AMR varied by *Salmonella* Infantis cluster and by isolation source; 73% of poultry isolates were multidrug resistant, compared with 35% of human isolates. This finding correlated with the presence of the pESI megaplasmid; 71% of poultry isolates contained pESI, compared with 32% of human isolates. This study provides key information for public health teams engaged in reducing the spread of this pathogen.

Nontyphoidal *Salmonella* infections place a large burden on public health; an estimated 79 million cases of foodborne nontyphoidal *Salmonella* infection occurred in 2010 (*1*). *Salmonella enterica* subspecies *enterica* serovar Infantis is becoming an increasingly prevalent serovar globally. A 167% increase in human infections was observed in the United States during 2001–2016 (*2*), and in European Union member states, Infantis is the predominant serovar isolated from broiler flocks and broiler meat, accounting for 56.7% of *Salmonella* isolates from broiler meat in 2018 (*3*,*4*). Higher levels have been observed in Japan, at 72.2% of isolates from ground chicken, and levels of 84% were seen in broilers in Ecuador (*5*,*6*).

Antimicrobial resistance (AMR) in *Salmonella* Infantis varies by location; in South Africa only 13.4% of 387 *Salmonella* Infantis isolates from humans had AMR (*7*). Conversely, in 2016 in European Union member states, 70% of *Salmonella* Infantis isolates from broiler meat were multidrug-resistant (MDR) (*8*). Of particular concern is the emergence of extended β-lactamases (ESBLs), such as the *bla*_CTX-M-65_ gene, which has been reported in *Salmonella* Infantis from Ecuador, Peru, Switzerland, the United Kingdom, and the United States (*9*–*13*). The pESI megaplasmid has been found to be responsible for these high levels of AMR because it confers resistance to trimethoprim, streptomycin, sulfamethoxazole, and tetracycline; ESBLs have also been found to be carried by some pESI variants (*10*,*11*,*14*). Originally identified in Israel, pESI-like plasmids have since been reported in multiple countries (*14*–*19*).

*Salmonella* Infantis has a polyphyletic population structure consisting of 2 eBurst Groups (eBG), eBG31 and eBG297, which differ by 5–7 multilocus sequence typing alleles (*16*). The dominant eBG (single locus variants around a central sequence type) globally is eBG31; eBG297 consisted of just 0.7% of *Salmonella* Infantis isolates in Enterobase on August 9, 2021 (*20*). However, higher levels (32%) of eBG297 have been reported in South Africa (*7*).

The population structure of *Salmonella* Infantis has been studied on a limited scale; whole-genome sequencing analysis of 100 *Salmonella* Infantis isolates from multiple continents and sources found no clustering by geographic location (*16*). *Salmonella* Infantis isolates were found to cluster by isolation source from human samples and chicken meat samples in Japan (*21*), by *bla*_CTX-M-65_ presence in human and animal strains in the United States and Italy (*11*), and by pESI presence in human and poultry isolates from Switzerland (*10*).

Although MDR *Salmonella* Infantis is an emerging public health concern, no large-scale population structure study of this pathogen has been performed. Because eBG297 isolates have been analyzed in depth (*7*), our aim was to determine the global population structure of eBG31 from a One Health perspective, investigating whether population structure is associated with isolation source, location, MDR properties, or pESI presence.

## Methods

Our eBG31 collection contained 5,284 isolates, sourced from the UK Health Security Agency (UKHSA), the National Institute for Communicable Diseases of South Africa, the Animal and Plant Health Agency, GenBank, and Enterobase (*20*) (Appendix 1). The collection contained strains isolated from 74 countries and spanned 4 decades and consisted of strains isolated during 1989–2020. The isolates were grouped into 8 sources: animal feed, human, environmental, food, other animals, poultry, poultry products, and unknown.

The whole-genome consensus FASTA sequences were grouped into clusters where all sequences in each cluster were <*n* single-nucleotide polymorphisms (SNPs) from another member. We generated a core SNP phylogeny of representatives of 25-SNP clusters; we identified clusters using fastbaps and used treedater to date the phylogeny (*22*–*25*). We used the ARIBA tool with the resfinder and plasmidfinder databases to screen for AMR determinants and plasmid presence; pESI was identified separately as described in Mattock et al. (*7*,*26*–*28*).

Ethical approval for the detection of gastrointestinal bacterial pathogens from fecal specimens, or the identification, characterization, and typing of cultures of gastrointestinal pathogens submitted to the Gastrointestinal Bacteria Reference Unit was not required because it is covered by UKHSA’s surveillance mandate. Ethical approval for all laboratory-based surveillance and research activities was obtained from the Human Research Ethics Committee, University of the Witwatersrand, Johannesburg, South Africa (protocol reference nos. M060449 and M110499) by the Centre for Enteric Diseases, National Institute for Communicable Diseases. Ethical approval for characterization of the isolates was not required because of the surveillance mandate of Animal and Plant Health Agency.

## Results

### Demographics

We included isolates from a multitude of sources in the eBG31 collection. Most (60%, 3,150) of the 5,284 isolates were isolated from humans and associated with either noninvasive infections (samples from stool and urine), or invasive infection (samples from blood and cerebrospinal fluid). A further 6% (300) were from poultry and 13% (684) from poultry products, which included samples from poultry meat, eggs, and processed meals containing poultry meat. Isolates from other animals made up 6% (321) of the collection, 7% (390) were from food, 1% (74) from animal feed, and 5% (268) environmental, such as water, farm swab, and soil samples. A total of 97 isolates had no stated isolation source.

**Figure 1:**
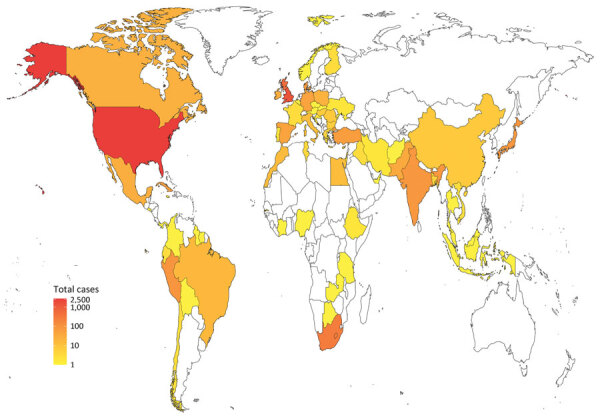
Heatmap indicating the number of isolates included in the dataset from each country in One Health–focused analysis.

The number of isolates increased temporally until 2018; this increase was caused by isolates from public databases being included until February 2018 (Appendix 1 Figure 1). Only strains isolated by the UKHSA were included after that time. When categorized by continent, 54% (2,861) were from North America, 31% (1,642) from Europe, 6% (316) from Africa, 6% (312) from Asia, and 2% (128) from South America; origins were unknown for 0.47% (25). The United States contributed the largest number of isolates (n = 2,719) followed by the United Kingdom (n = 1,326) (Table; Figure 1).

**Table:** Source group and country of isolates in One Health–focused analysis of *Salmonella enterica* serovar Infantis*

Country	Source group							
	Food	Environmental	Animal feed	Human	Poultry	Poultry products	Other animals	Unknown
Canada			4	21		1		
Cyprus								7
Denmark	18				1		36	11
Germany	16	7	8	1			15	1
Hungary				1				6
Japan		9		32	19	26		
Romania				2				6
South Africa		6		266				1
United Kingdom	75	52		1016	98	26	31	28
United States	234	185	57	1254	161	601	227	
Other	44	9	5	557	20	26	12	11
Unknown	3				1	4		26
Total	390	268	74	3150	300	684	321	97

*The number of isolates from each source group and the country of isolation were filtered to include countries that comprised >5% of >1 source group.

### Population Structure

At the sequence type (ST) level, most eBG31 isolates (99%, 5,205) belonged to ST32. The second most common was ST2283; 36 isolates belonged to this ST, all from Europe (17 from humans, 10 from other animals, 5 from food, and 4 from environmental samples). The third most common was ST2146 with 26 isolates, all from North America (22 were from environmental samples, 3 from food, and 1 from a clinical sample). The 13 remaining STs were found in <3 isolates.

**Figure 2:**
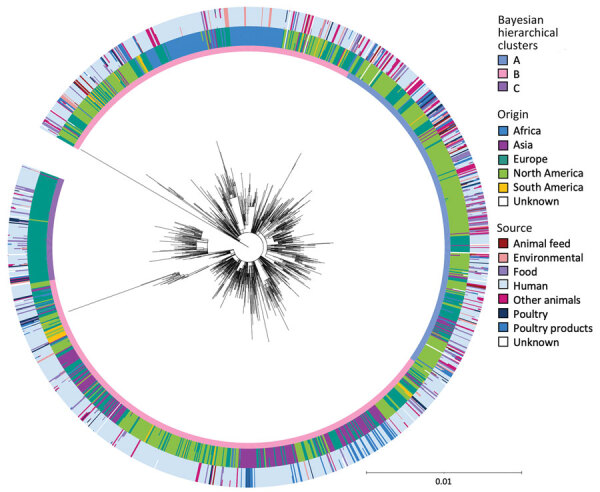
Core single-nucleotide polymorphism maximum-likelihood phylogeny of 1,288 representatives of 5,283 isolates in One Health–focused analysis of emerging multidrug-resistant pathogen *Salmonella enterica* serovar Infantis. The inner ring around the phylogeny is annotated with the Bayesian hierarchical clusters found by fastbaps. Cluster A consists of 348 representatives of 1,624 isolates; cluster B consists of 831 representatives of 3,283 isolates; and cluster C consists of 109 representatives of 376 isolates. The outer rings show the percentage of isolates in each 25-SNP cluster that were from each continent and source. Isolate origin was identified for Africa (n = 316), Asia (n = 312), Europe (n = 1,641), North America (n = 2,861), and South America (n = 128); the origin of the remaining isolates was unknown (n = 25). Sources were animal feed (n = 74), environmental (n = 268), food (n = 390), human (n = 3,149), other animals (n = 321), poultry (n = 300), poultry products (n = 684), and unknown (n = 97).

**Figure 3:**
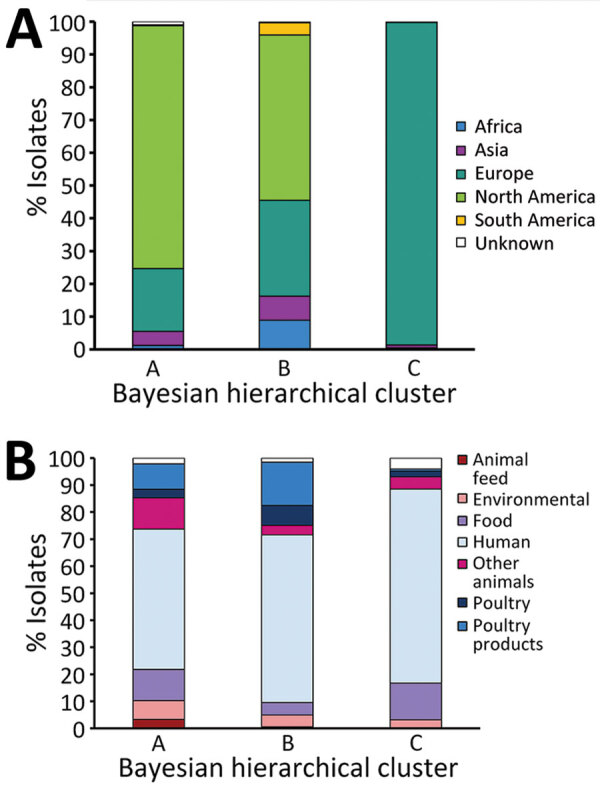
Source and continent composition of fastbaps clusters in study of One Health perspective of emerging multidrug-resistant pathogen *Salmonella enterica* serovar Infantis. A) Percentage of isolates from each continent in clusters A (n = 1,624), B (n = 3,283), and C (n = 376). B) Percentage of isolates from each source group in clusters A (n = 1,624), B (n = 3,283), and C (n = 376).

We identified 3 clusters of 250 SNPs; 1 contained just SRR8114924. For 50-SNP clusters, there were 408; for 25-SNP cluster, 1,288; for 10-SNP clusters, 2,876; and for 5-SNP clusters, 3,917. In a core SNP maximum-likelihood phylogeny of a member of each 25-SNP cluster, representing 5,283 eBG31 isolates (Figure 2; Appendix 1 Figure 2), Bayesian hierarchical clustering identified 3 clusters. Cluster A contained 348 sequences, representing 1,624 isolates (Figure 2, blue); cluster B had 831 sequences, representing 3,283 isolates (Figure 2, pink); and cluster C, which diverged from within cluster B, contained 109 sequences, representing 376 isolates (Figure 2, purple). When annotated by ST, the phylogeny was dominated by ST32 (Appendix 1 Figure 3); 99% (1,269/1,288) of the 25-SNP clusters were exclusively ST32. The three 25-SNP clusters containing the ST2283 isolates clustered together in cluster C, and another 3 clusters comprising the ST3815 isolates clustered in cluster A.

In contrast to previous reports, a geographic signal was visible in the clustering of isolates in the phylogeny. Cluster A mainly consisted of North American isolates (Figure 3, panel A), of which 98% (1,180/1,203) were from the United States. Cluster B also contained a large percentage of North American isolates (50%, 1,657/3,238), but higher percentages of isolates from all other continents were observed. Conversely, cluster C consisted almost exclusively of European isolates, most of which were isolated in the United Kingdom (80%, 297/370).

The predominant isolation source in each of the clusters was humans (Figure 3, panel B). Isolates from poultry and poultry products were most often found in cluster B. However, isolates from other animals and animal feed made up a larger proportion of cluster A than the other clusters.

**Figure 4:**
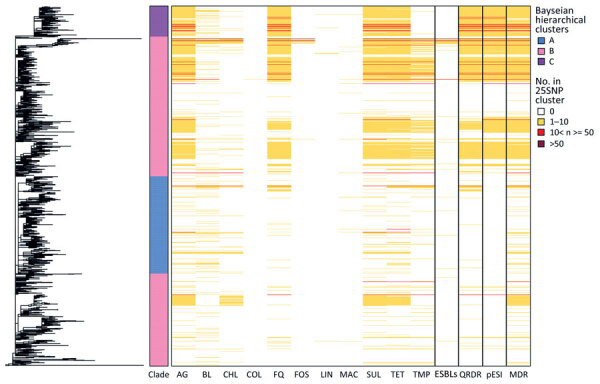
Phylogeny and heatmap of antimicrobial resistance and pESI in study of One Health perspective of emerging multidrug-resistant pathogen *Salmonella enterica* serovar Infantis. Heatmap shows the number of isolates in each 25 single-nucleotide polymorphism representative cluster (n = 1,288) of the eBG31 maximum-likelihood phylogeny with genes conferring resistance to antimicrobial drugs. Fastbaps clade and the number of isolates with MDR, ESBLs, mutations in the QRDR conferring resistance to fluoroquinolones and pESI presence are also shown. AG, aminoglycosides; BL, β-lactams; CHL, chloramphenicol; COL, colistin; ESBLs, extended β-lactamases; FQ, fluoroquinolones; FOS, fosfomycin; LIN, lincosamides; MAC, macrolides; MDR, multidrug-resistant; SUL, sulphonamides; TET, tetracyclines; TMP, trimethoprim.

Minimal clustering by year was observed in the phylogeny (Appendix 1 Figure 4). The most common year range in each cluster was 2016–2020, representing 48% (785/1,624) of cluster A, 51% (1,664/3,238) of cluster B, and 67% (253/376) of cluster C. The earliest date of isolation varied; cluster B was the oldest with an isolate from 1989. Cluster A’s oldest isolates were from 1996, and cluster C appeared more recently; its oldest isolate was from 2007. The time of the most recent common ancestor was calculated using relaxed clock dating of the phylogeny and estimated to be 1946; cluster A diverged in 1982 and cluster C in 1987.

We calculated the pairwise nucleotide distance between each whole-genome consensus FASTA to show the diversity within and between groups of isolates. Clusters B and C had the lowest median pairwise nucleotide distance of 191 (range 32–1,743); higher values were observed between clusters A and B (304, range 26–1,983) and clusters A and C (428, range 80–1,410). When comparing within isolation sources, strains from poultry products had the lowest median pairwise nucleotide distance at 145 (range 0–1,249) and human isolates the largest at 241 (range 0–2,060). The median pairwise nucleotide distance between human isolates and other sources ranged from 215 for environmental isolates (range 1–2,004) to 259 for poultry isolates (range 0–2,039). Poultry isolates had a similar median nucleotide distance to environmental isolates (251, range 4–1,822); a larger distance was observed between poultry isolates and those from other animals (310, range 4–1,817). The largest median pairwise nucleotide distance between source groups was poultry and animal feed at 334 (range 5–1,614). Low median pairwise nucleotide distances were observed within isolates from South America (44, range 0–920) and North America (159, range 0–1,883); the largest distance within isolates from a continent was Africa at 492 (range 0–1,885). Isolates from Africa also had the largest median pairwise nucleotide distances of all continents; distance was 395 (range 15–2,059) with North America and 422 (range 23–1,639) with South America.

### AMR in *Salmonella* Infantis

In this collection, 44% (2,327/5,284) of the isolates contained ≥1 AMR gene; most of those (40%, 2,101/5,284) were MDR. Genes encoding AMR were identified in isolates throughout the phylogeny (Figure 4); 46.7% (602/1,288) of the 25-SNP clusters contained an isolate with AMR. Some 25-SNP clusters contained large numbers of isolates with AMR, such as 1 in cluster B that contained 734 isolates, of which 727 were MDR. A common resistance profile was visible in 25-SNP clusters across the phylogeny: 27.7% (357/1,288) contained an isolate with resistance to aminoglycosides, fluoroquinolones, sulphonamides, and tetracyclines (AFST). Of those, 56.9% (203/357) had an isolate with putative trimethoprim resistance, and 99.7% (356/357) contained a mutation in the quinolone-resistance determining region. The percentage of isolates with this resistance profile varied between the clusters: 1% (16/1,624) in cluster A, 43.5% (1,407/3,283) in cluster B, and 84% (316/376) in cluster C. The percentage of MDR isolates differed between the clusters; 7% (114/1,624) of isolates from cluster A were MDR, 50.5% (1,657/3,283) of isolates from cluster B were MDR, and 87.8% (330/376) from cluster C were MDR. More isolates were positive for ESBLs in cluster B (20.5%, 672/3,283) than in clusters A (0.2%, 4/1,624) and C (0%).

We observed variation in the distribution of AMR between isolation sources. Isolates from animal feed had the lowest amount of AMR; 4% (3/74) were predicted to have the AFST resistance profile. Higher levels of AMR were predicted in human isolates; 29% (913/3,150) had the AFST resistance profile, and 60% (552/913) of those also had trimethoprim resistance genes. Substantially more AMR was present in poultry and poultry product isolates: 61% (183/300) of poultry isolates and 64% (438/684) of poultry product isolates had the AFST resistance profile. β-lactam and chloramphenicol resistance was more common in poultry product isolates (43% [296/684] for β-lactam and 44% [302/684] for chloramphenicol) than in poultry isolates (21% [64/300] for β-lactam and 22% [65/300] for chloramphenicol). ESBLs were identified in 10% (312/3,150) of human isolates, 19% (58/300) of poultry isolates, and 40% (272/684) of isolates from poultry products. We observed multidrug resistance in 4% (3/74) of isolates from animal feed, 14% (38/268) of environmental isolates, 32% (123/390) of food isolates, 35% (1,115/3,150) of human isolates, 21% (67/321) of isolates from other animals, 73% (218/300) of poultry isolates, and 73% (501/684) of poultry product isolates.

AMR profiles also varied by continent of isolation. The lowest levels of AMR were observed in isolates from Africa, of which 4% (14/316) had the AFST resistance profile, compared with 27% (763/2,861) of isolates from North America, 42% (692/1,642) of isolates from Europe, 55% (172/312) of isolates from Asia, and 76% (97/128) of isolates from South America. ESBLs were present in 0.3% (1/316) of isolates from Africa, 3% (9/312) of isolates from Asia, 4% (65/1,642) of isolates from Europe, 19% (531/2,861) of isolates from North America, and 55% (70/128) of isolates from South America. The percentage of MDR isolates was 20% (63/316) for Africa, 31% (891/2,861) for North America, 48% (793/1,642) for Europe, 80% (248/312) for Asia, and 81% (104/128) for South America.

The proportion of isolates with AMR fluctuated throughout the study period and trended upwards in the last 15 years of the collection period (Appendix 1 Figure 5). The earliest isolate in the collection (from 1989) was predicted to be resistant to 6 antimicrobial classes. AMR to aminoglycosides, sulphonamides, and tetracyclines were consistently the most common and appeared to follow a similar trend; after 2012, similar levels of AMR to fluoroquinolones were also present.

### Plasmids in *Salmonella* Infantis

As observed with AMR, <50% (47%, 2502/5284) of *Salmonella* Infantis isolates contained a plasmid. Some of the most common types included IncA/C (n = 103), IncI1 (n = 251), and IncX1 (n = 65). As expected, pESI was the prevailing plasmid type, present in 36% (1,912/5,284) of *Salmonella* Infantis isolates. Low levels of IncA/C were observed in all isolation sources except animal feed; the highest level was just 4% (14/321) of other animal isolates, 2% of both human (64/3,150) and poultry product (15/684) isolates, and 0.6% (2/300) of poultry isolates. Incl1 was most common in other animal isolates at 9% (28/321) and IncX1 in human isolates (2%, 52/3,150). IncI1-positive isolates were found in all continents throughout the study period, mainly from humans (n = 184); IncX1 was observed in Asia, Europe, and North America.

The presence of pESI-like plasmids was observed in 71% (213/300) of poultry isolates, 71% (486/684) of isolates from poultry products, 32% (992/3,150) of human isolates, 4% (3/74) of animal feed isolates, 10% (31/321) of isolates from other animals, 31% (120/390) of food isolates, and 11% (30/268) of environmental isolates. Presence also varied by geographic location: the lowest percentage of pESI-positive isolates was from Africa at 4% (12/316), followed by North America at 28% (808/2,861), Europe at 47% (770/1,642), Asia at 71% (222/312), and South America at 77.3% (99/128). The earliest isolation of pESI in this collection was in 4 human isolates from Japan in 1999 (DRR022718, DRR022719, DRR022720, DRR022754). Although the common resistance profile AFST was distributed throughout the eBG31 phylogeny, cluster A lacked any pESI; most of cluster C contained pESI (99.7%, 375/376), and 46.8% (1,537/3,283) of cluster B contained pESI (Figure 4).

## Discussion

In our large core SNP analysis of *Salmonella* Infantis, we determined that the global population structure of eBG31 consists of 3 clusters with varying isolation sources and levels of AMR. As observed previously, the dominant ST in eBG31 was ST32, comprising 99% of the isolates (*29*–*32*). The other STs were not observed in multiple continents, suggesting that those STs have emerged in specific areas but have not spread globally. A strong geographic signal was identified in the eBG31 phylogeny (Figure 2); we hypothesize that cluster B is an ancestor of the 2 other clusters, containing more genetic and geographic diversity in isolates from all continents, and we therefore designate this the global *Salmonella* Infantis cluster. Cluster A, estimated to have diverged from cluster B in 1982, mainly consisted of isolates from North America and hence is named the North American cluster. Cluster C, which diverged from cluster B in 1987, was dominated by isolates from Europe and is thus named the European cluster. This designation differs, perhaps because of our larger number of isolates, from Gymoese et al. (*16*), who found no geographic signal when examining isolates from 5 continents, and Alba et al. (*33*), who reported little clustering by location or source. Some clustering by country of isolation was described in Acar et al. (*34*); however, because that clustering was between isolates from the same region in Turkey, the contribution to global clustering was not clear. Nucleotide distances relative to the reference showed that the African eBG31 isolates were both the most diverse and the most distant to isolates from other continents; our previous work identified that an increased proportion of isolates belonged to eBG297, and this study affirms that the African *Salmonella* Infantis population differs from that observed elsewhere (*7*).

Because most eBG31 isolates were from human sources, the phylogeny was dominated by this source. Cluster C in particular contained lower numbers of environmental, poultry, and poultry product isolates; that was possibly caused by bias in data sampling because the cluster contains strains from UKHSA that were isolated after the cutoff for inclusion from Enterobase. Most of those strains were from humans, as environmental sampling tends to be performed in association with an outbreak. Although cluster C contained strains isolated as early as 2007, it contained many of the newer strains isolated during 2018–2020; this group could represent an emerging clade of *Salmonella* Infantis. The nucleotide distance between source groups, relative to the reference, identified the least diversity in the poultry product isolates and the greatest diversity between poultry/poultry products and animal feed or other animal genomes. This finding could indicate a reduction of adaptation in poultry hosts and suggest that the different niches have encouraged adaptation; the reduced distance between poultry isolates could, however, be attributed to the large number of North American poultry isolates reducing the median (e.g., 519/734 of strains in the 25-SNP cluster with the representative SRR2537092 were from poultry, and 724 isolates in that cluster were from North America).

As described in many other studies, the *Salmonella* Infantis isolates in this project were associated with high levels of putative AMR (*8*,*18*,*35*,*36*). MDR genotypes were detected in 40% of the *Salmonella* Infantis isolates; their presence was notable in isolates from poultry and poultry products, in which 73% were MDR. In comparison, just 14% of environmental isolates, 21% of isolates from other animals, 32% of food isolates, and 35% of human isolates were MDR (Appendix 2 Table 1); this finding could indicate that the source of human infection was the nonpoultry sources with similar levels of AMR. Although that hypothesis has been suggested in Slovenia, where most broiler isolates clustered separately from human isolates (*32*), many incidences of human outbreaks associated with poultry have been reported (*21*,*37*,*38*). Both environmental and poultry sources could be contributing to human cases, but the pESI-positive strain circulating in the poultry industry could also have a selective disadvantage to causing infection in humans, leading to less frequent observation of those strains.

Levels of AMR varied by continent; the lowest levels were observed in Africa (20% of strains were MDR) and the highest were observed in South America (81%). The higher levels in South America concurs with other reports that found all but 1 isolate tested in Ecuador were MDR and observed multiple drug resistance profiles in *Salmonella* Infantis strains from Chile (*38*,*39*). AMR fluctuated temporally, increasing in the last 15 years of the collection period. The proportion of isolates with resistance to aminoglycosides, sulphonamides, and tetracyclines followed a similar trend, joined by fluoroquinolones after 2012. This trend could be attributed to pESI, which carries resistance genes for those antimicrobial drugs. Similarly, we noted an association between pESI presence and resistance to aminoglycosides, fluoroquinolones, sulphonamides, and tetracyclines; multidrug resistance; and mutations in the quinolone-resistance determining region (Figure 4). The pESI backbone has been confirmed to carry *aadA1*, *sul1*, and *tetA* (*14*,*19*), illustrating that pESI is a strong driver of AMR in the *Salmonella* Infantis population, which supports the suggestion of Alba et al. (*33*) that pESI acquisition could be the decisive factor in the spread of the serovar throughout Europe. Of note, cluster C, the clade we suspect is an emerging dominant strain in Europe, is dominated by AMR and pESI, but cluster A, the North American cluster with relatively low levels of MDR strains (7%), lacked any pESI-positive isolates. The pESI-like plasmid was present in 808 North American isolates in this dataset, but they belonged to either cluster B or C. This finding concurs with previous research that reported 2 clades within the US *Salmonella* Infantis population, 1 with and 1 without pESI, and suggests that 2 groups of *Salmonella* Infantis are circulating in North America: 1 associated with MDR strains and pESI, and the other endemic to North America and not carrying pESI (*40*). The presence of pESI-like plasmids has recently been identified in *Salmonella* serovars Agona, Muenchen, Schwarzengrund, and Senftenberg; the increased virulence of pESI-positive isolates and transmissibility of this plasmid within the *Salmonella* Infantis global population and to other *Salmonella* serovars is a grave public health concern (*41**–**43*).

In conclusion, most *Salmonella* Infantis isolates fall within eBG31, which consists of 3 clusters: a North American cluster (cluster A), a European cluster (cluster C), and an ancestral but still extant global cluster (cluster B). Isolates from Africa were genetically more diverse and distant from isolates from the other continents, further confirming previous work that identified a distinct population structure in *Salmonella* Infantis in South Africa. Using a One Health approach, we observed high levels of AMR in poultry and poultry products, highlighting the need to reduce the levels of this pathogen in poultry production premises and encouraging the development and use of a vaccine against *Salmonella* Infantis in poultry. Finally, pESI-like plasmids were shown to be a major driver for AMR in the global *Salmonella* Infantis population, posing a major threat to public health.

Appendix 1 Additional information about A One Health perspective on *Salmonella enterica* serovar Infantis, the emerging human multidrug-resistant pathogen

Appendix 2 Additional data used in study of A One Health perspective on *Salmonella enterica* serovar Infantis, the emerging human multidrug-resistant pathogen
