# Supplementary material for: A One Health Perspective on Salmonella enterica Serovar Infantis, an Emerging Human Multidrug-Resistant Pathogen
Source: Emerg Infect Dis. 2024 Apr;30(4):701–10. doi: 10.3201/eid3004.231031 (PMC10977846; doi:10.3201/eid3004.231031)
Supplement: Appendix 1 — Additional information about A One Health perspective on Salmonella enterica serovar Infantis, the emerging human multidrug-resistant pathogen [file 23-1031-Techapp-s1.pdf]

Article DOI: https://doi.org/10.3201/eid3004.231031

EID cannot ensure accessibility for supplementary materials supplied by authors. Readers who have difficulty accessing supplementary content should contact the authors for assistance.

A One Health Perspective on *Salmonella enterica* Serovar Infantis, the Emerging Human Multidrug-Resistant Pathogen

Appendix 1

Methods

Isolate selection

Enterobase was searched on the 19th of February 2018, and all isolates belonging to eBG31 with sequence data available in the short read archive (SRA) were included; due to the computational cost of including more sequences we chose to not include isolates after this cutoff (*1*). All UK Health Security Agency (UKHSA) eBG31 sequence data reported by 31st of December 2020 was downloaded from the *UKHSA* Pathogens *BioProject* at NCBI (PRJNA248792). Sequence data for 62 Animal and Plant Health Agency (APHA) *S.* Infantis isolates were downloaded from the SRA on 28th of May 2019.

DNA of *S.* Infantis isolates was shared for sequencing by the APHA, UKHSA and National Institute of Communicable Diseases (NICD), South Africa. The APHA isolates included had been collected for surveillance up until March 2018. Strains, isolated between 2000 and 2014, were selected from the UKHSA culture store; ensuring that there was an even distribution by time and, if applicable, continent of travel. Strains isolated between 2004 and 2016 were selected from the NICD culture store for sequencing.

The metadata available was used to stratify the isolates into the following source groups: human, food, poultry, poultry products, animal feed, other animals, environmental and unknown (Appendix 2 Table 1). All clinical isolates and those with metadata on patient age or gender were

classified as human. Isolates from chickens, ducks, turkeys and quail were categorised as poultry. All strains from poultry products, such as eggs and chicken meat, were grouped together. Isolates from all other animals were grouped into ‘other animals’ and non-poultry animal products and other food samples were grouped into ‘food’. All samples from animal feed were grouped and farm swabs, soil, water, air and sewage were designated environmental. Isolates missing source information to assign to these groups were classed as unknown. For the UKHSA isolates with travel history information, the continent of travel was used to designate the continent of isolate origin. The world map plot was generated with R (v.4.1.0) and the package ggplot2 (v.3.3.6) (*2*,*3*).

Whole Genome Sequencing

DNA from the NICD isolates was extracted using QIAamp DNA Mini Kit (Qiagen) and sequenced as described in Mattock *et al*., 2021 (*4*); libraries were prepared using either Illumina Nextera XT library preparation kits or using a custom library preparation method described in Rasheed *et al*., 2020 (*5*). Twelve NICD isolates not used in Mattock *et al.*, 2021 are included here and a library was prepared using one of the aforementioned methods and sequenced on an Illumina NextSeq 500.

The DNA of the UKHSA samples was extracted using the Qiasymphony DSP DNA Midi Kit (Qiagen), following a protocol described in Nair *et al*., 2016 (*6*). The historical UKHSA samples, described in Lee *et al*., 2021, were also sequenced using either Illumina Nextera XT or the custom library preparation methods (*7*). Two additional historical UKHSA samples were included and sequenced using the custom library preparation method. The contemporary UKHSA samples were prepared using the Illumina Nextera XT protocol and sequenced on an Illumina HiSeq 2500 instrument.

DNA from the APHA isolates was extracted using the MagMAX CORE Nucleic Acid Purification Kit (ThermoFisher) with a KingFisher Flex Purification System (ThermoFisher). The DNA was then sequenced using the custom library preparation method and an Illumina NextSeq 500.

Phylogenetic analysis

All the sequence data generated by UKHSA were trimmed using Trimmomatic (v0.27) with leading and trailing at <Q30 (*8*). All other sequence data were trimmed with Trimmomatic

(v0.36) and the same options. Sequence type and eBG were determined for each isolate using Metric-Oriented Sequence Typer (MOST) (v.1.0) with a UKHSA *Salmonella* database (*9*,*10*). The quality of borderline sequence typing was assessed using Tablet (*11*).

Using the Cloud Infrastructure for Big Data Microbial Bioinformatics (*12*), each sample was mapped and variant called against the eBG31 reference CP070301 (*7*), using Snippy (v.4.6.0) with the options minfrac 0.9 and 30 (*13*). Due to the large number of sequences included, it was not computationally possible to produce a phylogeny including all of the isolates. Therefore, the SNP distance between all of the consensus FASTAs produced by Snippy was calculated using snp-dists (v.0.7) (https://github.com/tseemann/snp-dists) with the -m option to output in the molten format. Using MCL (v14–137) and the abc option, Markov clustering was performed on the pairwise SNP distance matrix with the following SNP distance thresholds: 0-SNPs, 5-SNPs, 10-SNPs, 25-SNPs, 50-SNPs, 100-SNPs and 250-SNPs (*10*,*14*). This resulted in clusters where each cluster member was less than ‘n’ SNPs from another member. The smallest SNP threshold that it was possible to generate a phylogeny with was 25-SNPs; the first isolate in each 25-SNP cluster was chosen to be the representative for that cluster.

The eBG31 reference genome, CP070301, was screened for prophages using PHASTER (*7*,*15*). Four complete prophages were identified and masked by Snippy when producing an alignment of the 25-SNP cluster representatives. The whole genome alignment was used by Gubbins (v.2.4.1) to identify recombination which was then removed during core-SNP alignment generation (*16*). A core SNP maximum likelihood phylogeny of the 25-SNP cluster representatives, excluding the reference isolate, was constructed using RaxML (v.8.2.12), rooted to its most ancestral node and annotated using iToL (*17*,*18*). Hierarchical Bayesian clustering within the phylogeny was identified using fastbaps (v.1.0.5) with ape (v.5.3) and R (v.3.4.1), with the optimised symmetric prior and k.init at 5 (*2*,*19*,*20*). Pairwise SNP distances were calculated using the snp-dists output, with self against self comparisons excluded.

The treedater relaxed clock test was performed to determine support for using a strict clock; the strict clock was deemed the best approach (*21*). Treedater (v.0.5.0) was used with a strict clock and the dates ranges of the isolates in each 25-SNP cluster, excluding leaves with an unknown isolation date, to date the phylogeny. Phangorn (v.2.10.0) was used to identify the nodes where the clades diverged (*22*).

AMR and plasmid determination

ARIBA (v.2.10.1) was used with the ResFinder and Plasmidfinder databases (downloaded 17th January, 2022) to identify AMR determinants and plasmids (*23–25*). A database of *gyrA*, *gyrB*, *parC* and *parE*, from the *S.* Typhimurium LT2 reference, was used with ARIBA to identify mutations within the Quinolone Resistance Determining Regions (QRDR). MDR, resistance to three or more antimicrobial classes, was calculated using AMR genes identified by ARIBA and mutations in QRDRs. As aminoglycoside resistance in *Salmonella* is rarely conferred by *aac(6')-Iaa* it was excluded from all calculations (*26*). pESI presence was determined by mapping each sequence against a pseudomolecule of the eBG31 reference (CP070301) and pESI contigs (ASRF01000099-ASRF01000108) with SMALT (v.0.7.6) and calculating coverage with Samtools (v.1.5) (*27*,*28*). Heatmaps were generated using phytools (v.0.6) and data.table (v.1.11.8) (*29*,*30*).

The proportion of isolates containing each AMR determinant and plasmid from each metadata group was calculated in R (v.4.1.0) with the packages janitor (v.2.1.0), tidyr (v.1.1.3) and magrittr (v.2.0.1) (*31–33*). The heatmap was created in R with ape and gplots (v3.1.3) (*34*).

References

1. Alikhan N-F, Zhou Z, Sergeant MJ, Achtman M. A genomic overview of the population structure of *Salmonella.* PLoS Genet. 2018;14:e1007261. PubMed https://doi.org/10.1371/journal.pgen.1007261

2. The R Foundation. The R Project for Statistical Computing [cited 2021 May 12]. https://www.r-project.org/

3. ggplot2: elegant graphics for data analysis [cited 2022 May 10]. https://ggplot2.tidyverse.org

4. Mattock J, Smith AM, Keddy KH, Manners EJ, Duze ST, Smouse S, et al. Genetic characterization of *Salmonella* Infantis from South Africa, 2004–2016. Access Microbiol. 2022;4:acmi000371. PubMed https://doi.org/10.1099/acmi.0.000371

5. Rasheed F, Saeed M, Alikhan N-F, Baker D, Khurshid M, Ainsworth EV, et al. Emergence of resistance to fluoroquinolones and third-generation cephalosporins in *Salmonella* Typhi in Lahore, Pakistan. Microorganisms. 2020;8:1336. PubMed https://doi.org/10.3390/microorganisms8091336

6. Nair S, Ashton P, Doumith M, Connell S, Painset A, Mwaigwisya S, et al. WGS for surveillance of antimicrobial resistance: a pilot study to detect the prevalence and mechanism of resistance to azithromycin in a UK population of non-typhoidal *Salmonella.* J Antimicrob Chemother. 2016;71:3400–8. PubMed https://doi.org/10.1093/jac/dkw318

7. Lee WWY, Mattock J, Greig DR, Langridge GC, Baker D, Bloomfield S, *et al*. Characterization of a pESI-like plasmid and analysis of multidrug-resistant *Salmonella enterica* Infantis isolates in England and Wales. Microb Genom. 2021;10:000658. **PMID 34647862**

8. Bolger AM, Lohse M, Usadel B. Trimmomatic: a flexible trimmer for Illumina sequence data. Bioinformatics. 2014;30:2114–20. PubMed https://doi.org/10.1093/bioinformatics/btu170

9. Tewolde R, Dallman T, Schaefer U, Sheppard CL, Ashton P, Pichon B, et al. MOST: a modified MLST typing tool based on short read sequencing. PeerJ. 2016;4:e2308. PubMed https://doi.org/10.7717/peerj.2308

10. Chattaway MA, Dallman TJ, Larkin L, Nair S, McCormick J, Mikhail A, et al. The transformation of reference microbiology methods and surveillance for *Salmonella* with the use of whole genome sequencing in England and Wales. Front Public Health. 2019;7:317. PubMed https://doi.org/10.3389/fpubh.2019.00317

11. Milne I, Stephen G, Bayer M, Cock PJA, Pritchard L, Cardle L, et al. Using Tablet for visual exploration of second-generation sequencing data. Brief Bioinform. 2013;14:193–202. PubMed https://doi.org/10.1093/bib/bbs012

12. Connor TR, Loman NJ, Thompson S, Smith A, Southgate J, Poplawski R, *et al*. CLIMB (the Cloud Infrastructure for Microbial Bioinformatics): an online resource for the medical microbiology community. Microb Genom. 2016;2:e000086. **PMID 28785418**

13. Seemann T. Snippy: rapid haploid variant calling. 2015 [cited 2021 Feb 8]. https://github.com/tseemann/snippy

14. Enright AJ, Van Dongen S, Ouzounis CA. An efficient algorithm for large-scale detection of protein families. Nucleic Acids Res. 2002;30:1575–84. PubMed https://doi.org/10.1093/nar/30.7.1575

15. Arndt D, Grant JR, Marcu A, Sajed T, Pon A, Liang Y, et al. PHASTER: a better, faster version of the PHAST phage search tool. Nucleic Acids Res. 2016;44(W1):W16-21. PubMed https://doi.org/10.1093/nar/gkw387

16. Croucher NJ, Page AJ, Connor TR, Delaney AJ, Keane JA, Bentley SD, et al. Rapid phylogenetic analysis of large samples of recombinant bacterial whole genome sequences using Gubbins. Nucleic Acids Res. 2015;43:e15–15. PubMed https://doi.org/10.1093/nar/gku1196

17. Stamatakis A. RAxML version 8: a tool for phylogenetic analysis and post-analysis of large phylogenies. 2014;30(9):1312–3. https://doi.org/10.1093/bioinformatics/btu033 **PMID 24451623**

18. Letunic I, Bork P. Interactive tree of life (iTOL) v3: an online tool for the display and annotation of phylogenetic and other trees. Nucleic Acids Res. 2016;44(W1):W242-5. PubMed https://doi.org/10.1093/nar/gkw290

19. Tonkin-Hill G, Lees JA, Bentley SD, Frost SDW, Corander J. Fast hierarchical Bayesian analysis of population structure. Nucleic Acids Res. 2019;47:5539–49. PubMed https://doi.org/10.1093/nar/gkz361

20. Paradis E, Schliep K. ape 5.0: an environment for modern phylogenetics and evolutionary analyses in {R}. Bioinformatics. 2018. PubMed

21. Volz EM, Frost SDW. Scalable relaxed clock phylogenetic dating. Virus Evol. 2017;3:vex025. https://doi.org/10.1093/ve/vex025

22. Schliep KP. phangorn: phylogenetic analysis in R. Bioinformatics. 2011;27:592–3. PubMed https://doi.org/10.1093/bioinformatics/btq706

23. Hunt M, Mather AE, Sánchez-Busó L, Page AJ, Parkhill J, Keane JA, et al. ARIBA: rapid antimicrobial resistance genotyping directly from sequencing reads. Microb Genom. 2017;3:e000131. **PMID 29177089**

24. Carattoli A, Zankari E, García-Fernández A, Voldby Larsen M, Lund O, Villa L, et al. In silico detection and typing of plasmids using PlasmidFinder and plasmid multilocus sequence typing. Antimicrob Agents Chemother. 2014;58:3895–903. PubMed https://doi.org/10.1128/AAC.02412-14

25. Zankari E, Hasman H, Cosentino S, Vestergaard M, Rasmussen S, Lund O, et al. Identification of acquired antimicrobial resistance genes. J Antimicrob Chemother. 2012;67:2640–4. PubMed https://doi.org/10.1093/jac/dks261

26. Neuert S, Nair S, Day MR, Doumith M, Ashton PM, Mellor KC, et al. Prediction of phenotypic antimicrobial resistance profiles from whole genome sequences of non-typhoidal *Salmonella enterica.* Front Microbiol. 2018;9:592. PubMed https://doi.org/10.3389/fmicb.2018.00592

27. Wellcome Sanger Institute. SMALT [cited 2021 May 31]. https://www.sanger.ac.uk/science/tools/smalt-0

28. Li H, Handsaker B, Wysoker A, Fennell T, Ruan J, Homer N, et al.; 1000 Genome Project Data Processing Subgroup. The Sequence Alignment/Map format and SAMtools. Bioinformatics. 2009;25:2078–9. PubMed https://doi.org/10.1093/bioinformatics/btp352

29. Revell LJ. phytools: an R package for phylogenetic comparative biology (and other things). Methods Ecol Evol. 2012;3:217–23. https://doi.org/10.1111/j.2041-210X.2011.00169.x

30. Dowle M, Srinivasan A. data.table: Extension of 'data.frame.' R package version 1.11.8. 2018 [cited 2021 May 6]. https://cran.r-project.org/web/packages/data.table/data.table.pdf

31. Firke S. janitor: simple tools for examining and cleaning dirty data [cited 2021 Oct 06]. https://cran.r-project.org/web/packages/janitor/index.html

32. Wickham H, Vaughan D, Girlich M. tidyr. 2023 [cited 2021 Oct 9]. https://tidyr.tidyverse.org

33. Bache SM, Wickham H. magrittr. 2022 [cited 2021 May 5]. https://magrittr.tidyverse.org

34. Warnes G, Bolker B, Bonebakker L, Gentleman R, Huber W. gplots. 2022 [cited 2021 May 5]. https://github.com/talgalili/gplots

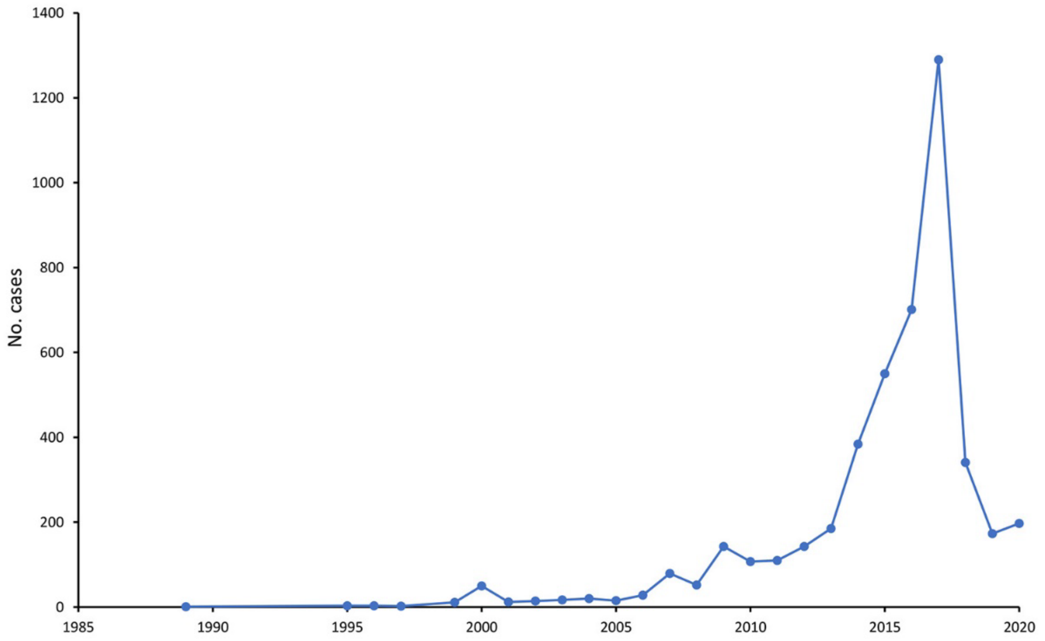

Appendix 1 Figure 1. Frequency of *S*. Infantis each year. Number of *S*. Infantis strains included in the dataset from 1989 to 2020.

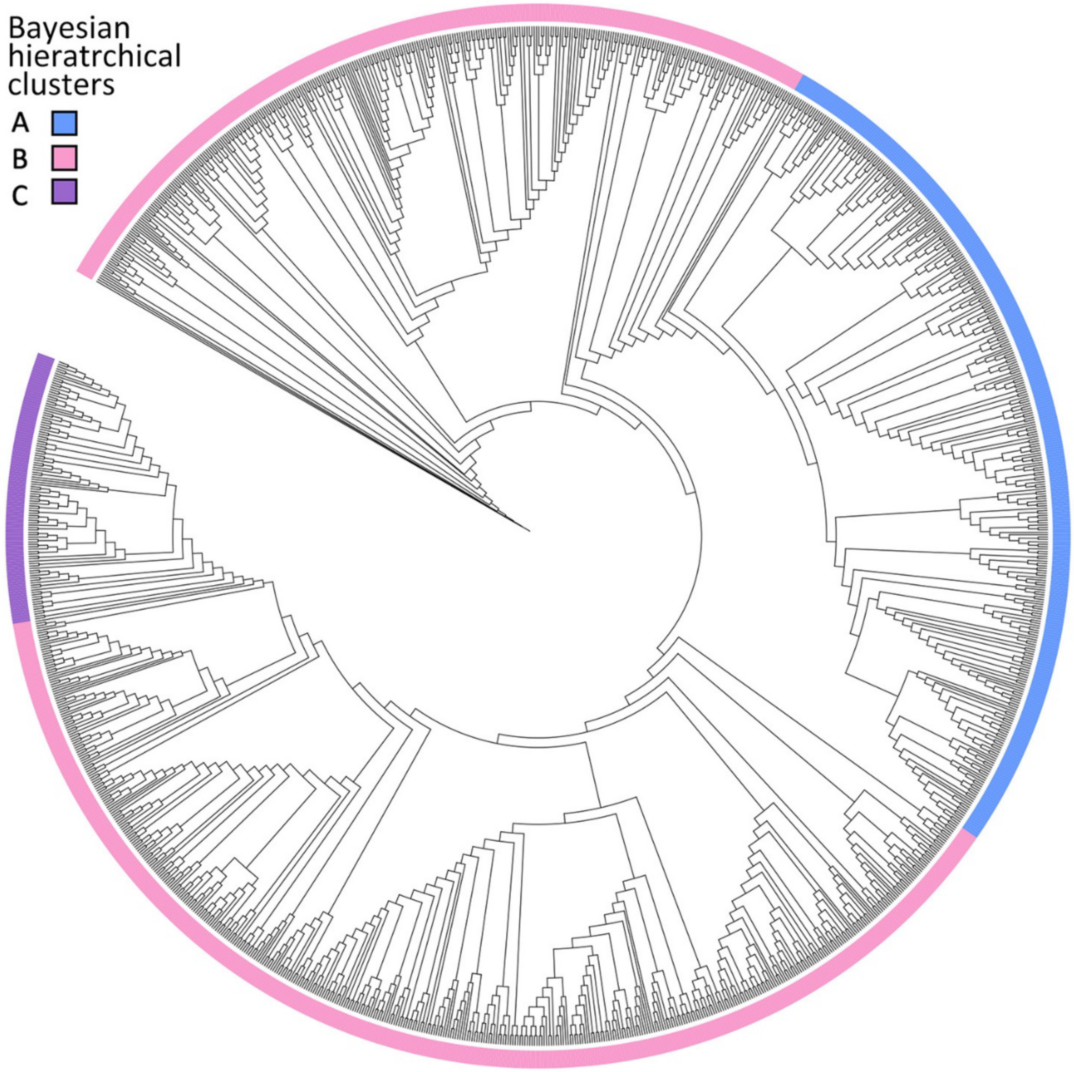

Appendix 1 Figure 2. Maximum likelihood cladogram of *S*. Infantis. Core SNP maximum likelihood cladogram of 1288 representatives of 5283 *S*. Infantis isolates. The ring around the cladogram is annotated with the Bayesian hierarchical clusters found by fastbaps. Bayesian hierarchical clusters: A, 348 representatives of 1624 isolates; B, 831 representatives of 3283 isolates and C 109 representatives of 376 isolates.

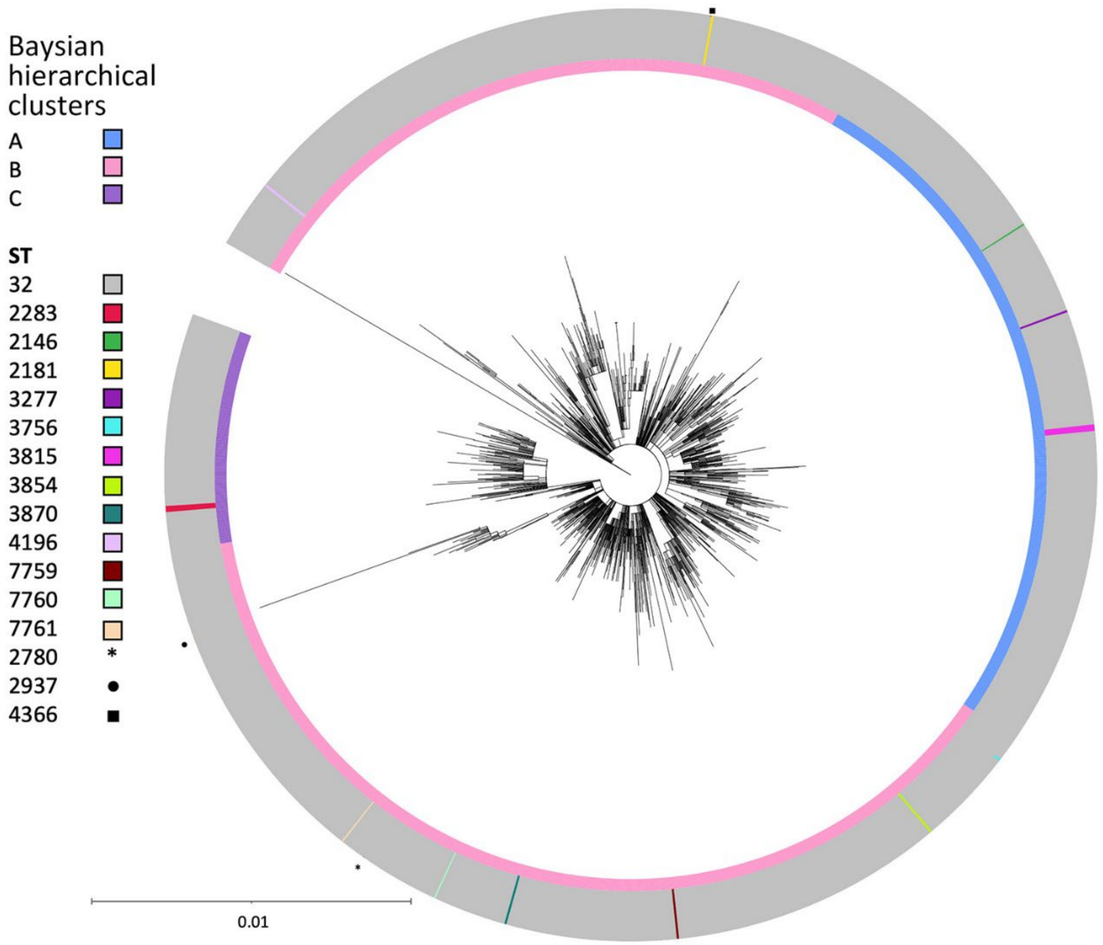

Appendix 1 Figure 3. Maximum likelihood phylogeny of *S*. Infantis with ST. Core SNP maximum likelihood phylogeny of 1288 representatives of 5283 *S*. Infantis isolates. The clusters in the phylogeny are annotated with the Bayesian hierarchical clusters found by fastbaps. The outer ring shows the percentage of isolates in each 25SNP cluster that belonged to each ST. Bayesian hierarchical clusters: A, 348 representatives of 1624 isolates; B, 831 representatives of 3283 isolates and C 109 representatives of 376 isolates. STs: 32 (n = 5204), 2283 (n = 36), 2780 (n = 1), 2181 (n = 1), 3756 (n = 2), 3277 (n = 1), 2146 (n = 26), 2937 (n = 2), 3815 (n = 3), 3870 (n = 1), 3854 (n = 1), 4196 (n = 1), 4366 (n = 1), 7759 (n = 1), 7760 (n = 1), 7761 (n = 1).

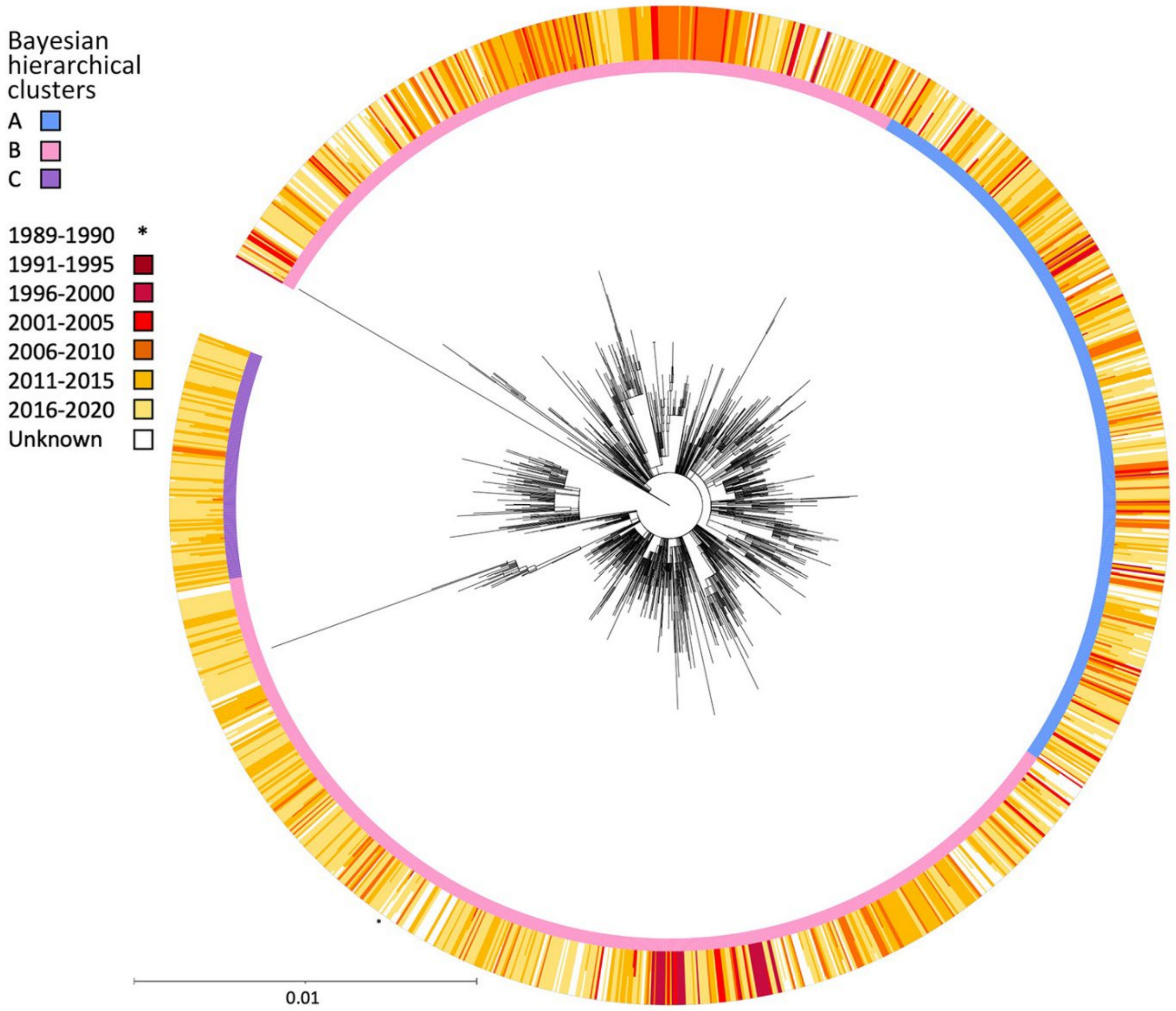

Appendix 1 Figure 4. Maximum likelihood phylogeny of *S*. Infantis with isolation year. Core SNP maximum likelihood phylogeny of 1288 representatives of 5283 *S*. Infantis isolates. The clusters in the phylogeny are annotated with the Bayesian hierarchical clusters found by fastbaps. The outer ring shows the percentage of isolates in each 25SNP cluster that were from each year group. Bayesian hierarchical clusters: A, 348 representatives of 1624 isolates; B, 831 representatives of 3283 isolates and C 109 representatives of 376 isolates. Years: 1989–1990 (n = 1), 1991–1995 (n = 3), 1996–2000 (n = 66), 2001–2005 (n = 78), 2006–2010 (n = 409), 2011–2015 (n = 1371), 2016–2020 (n = 2702), unknown (n = 653).

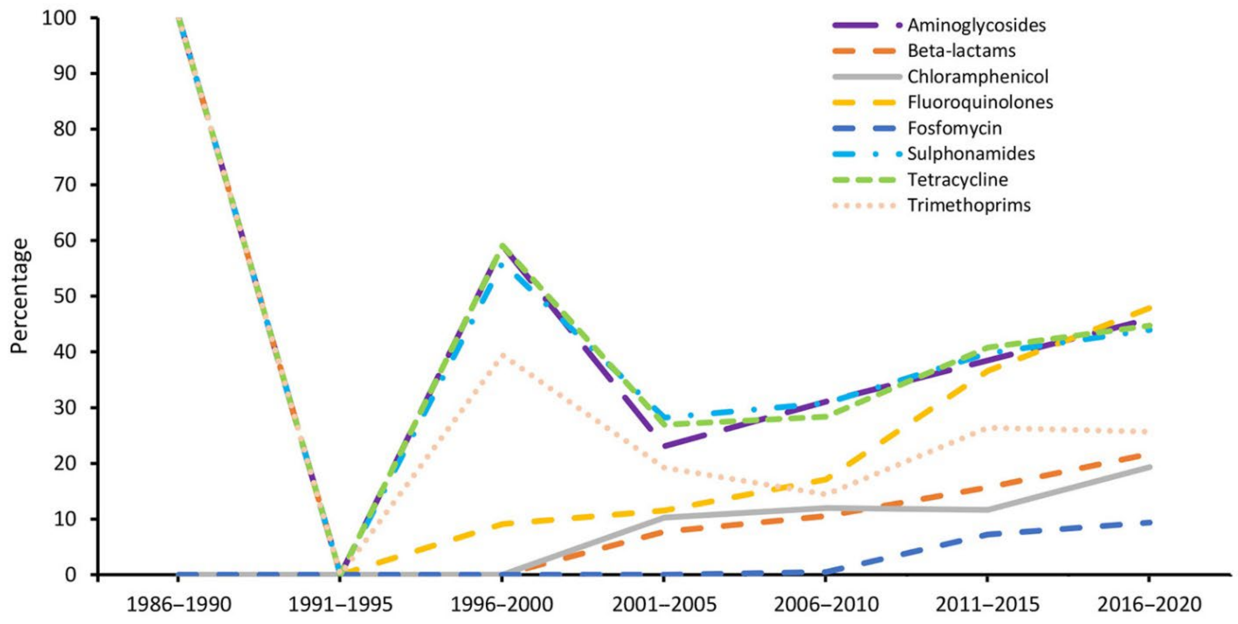

Appendix 1 Figure 5. AMR in *S*. Infantis each year. Proportion of *S*. Infantis isolates from each year with genes conferring resistance to the antimicrobial classes that had resistance in >5% of isolates: aminoglycosides, β-lactams, chloramphenicol, fluoroquinolones, sulphonamides, tetracyclines and trimethoprim. Years: 1989–1990 (n = 1), 1991–1995 (n = 3), 1996–2000 (n = 66), 2001–2005 (n = 78), 2006–2010 (n = 409), 2011–2015 (n = 1372), 2016–2020 (n = 2702), unknown (n = 653).
